# A Multi-Fluorescent DNA/Graphene Oxide Conjugate Sensor for Signature-Based Protein Discrimination

**DOI:** 10.3390/s17102194

**Published:** 2017-09-23

**Authors:** Shunsuke Tomita, Sayaka Ishihara, Ryoji Kurita

**Affiliations:** 1 Biomedical Research Institute, National Institute of Advanced Industrial Science and Technology, and DAILAB, 1-1-1 Higashi, Tsukuba, Ibaraki 305-8566, Japan; ishihara.sayaka@aist.go.jp; 2 Faculty of Pure and Applied Sciences, University of Tsukuba, 1-1-1 Tennodai, Tsukuba, Ibaraki 305-8573, Japan

**Keywords:** DNAs, nano-graphene oxide, proteins, multivariate analysis, biosensors

## Abstract

Signature-based protein sensing has recently emerged as a promising prospective alternative to conventional lock-and-key methods. However, most of the current examples require the measurement of optical signals from spatially-separated materials for the generation of signatures. Herein, we present a new approach for the construction of multi-fluorescent sensing systems with high accessibility and tunability, which allows generating protein fluorescent signatures from a single microplate well. This approach is based on conjugates between nano-graphene oxide (nGO) and three single-stranded DNAs (ssDNAs) that exhibit different sequences and fluorophores. Initially, the three fluorophore-modified ssDNAs were quenched simultaneously by binding to nGO. Subsequent addition of analyte proteins caused a partial recovery in fluorescent intensity of the individual ssDNAs. Based on this scheme, we have succeeded in acquiring fluorescence signatures unique to (i) ten proteins that differ with respect to pI and molecular weight and (ii) biochemical marker proteins in the presence of interferent human serum. Pattern-recognition methods demonstrated high levels of discrimination for this system. The high discriminatory power and simple format of this sensor system should enable an easy and fast evaluation of proteins and protein mixtures.

## 1. Introduction

The accurate identification of proteins is of critical importance for the understanding of a variety of biological processes and diseases [1,2]. Although the most frequently used lock-and-key approaches are successful [1,2,3], they often suffer from difficulties in obtaining specific receptors, such as antibodies and aptamers, for each target protein. In the past decade, signature-based sensing has emerged as a promising prospective alternative to lock-and-key specific recognition [4,5]. Signature-based sensors feature a group of “cross-reactive” materials that can interact in different ways with target proteins. Cross-reactive materials are usually integrated or complexed with reporter units (e.g., fluorescent and absorbent moieties) to give unique “multidimensional” optical signatures for individual proteins. A subsequent pattern-recognition of the thus-obtained signatures enables the accurate identification of proteins. Signature-based sensing has been successfully employed for the detection of proteins in dilute solutions [6,7,8,9,10,11,12,13,14,15,16,17] and in biological matrices [18,19,20,21,22,23,24,25,26,27]. However, most of the current examples require the measurement of optical signals from spatially-separated materials for the generation of signatures, e.g., in multiple wells of a microplate, which significantly limits the scope for applications that depend on a simple and rapid identification of proteins.

To address the aforementioned drawbacks, “multichannel” signature-based protein sensing systems have recently been developed, and these are based predominantly on two strategies: (i) the measurement of different optical properties from one type of material, and (ii) the measurement of a single optical property from one type of material or from a mixture of materials. The former so-called “lab-on-a-molecule” strategy uses different instruments to detect e.g., the fluorescence, phosphorescence, fluorescence polarization, and/or light-scattering intensity of materials, such as quantum dots [28,29,30] and graphene oxide (GO) [31]. Conversely, the latter strategy does not require multiple instruments for the readout of optical signatures [32], which significantly decreases the complexity and duration of the optical measurements. For example, three quantum dots with spectral resolvable fluorescence have been used to generate fluorescent signatures of proteins from a single microplate well [33]. Individual quantum dots can be synthetically modified with different functional groups, and subsequently be quenched simultaneously via conjugation with bromophenol blue. This sensing platform is capable of discriminating ten proteins and eight cell lines. Rotello et al. have applied a nanoparticle-based protein identification system [6,18,19] to multi-fluorescent sensing: quenched conjugates between gold-nanoparticles and three fluorescent proteins have been used for the detection of bacterial biofilms [34], mammalian cells [35,36], and drug-induced changes on cell surfaces [37]. However, these approaches still require laborious synthetic [33] or protein expression protocols [34,35,36,37], which represents a viable obstacle to adapt and extend this strategy to practical applications.

**Figure 1:**
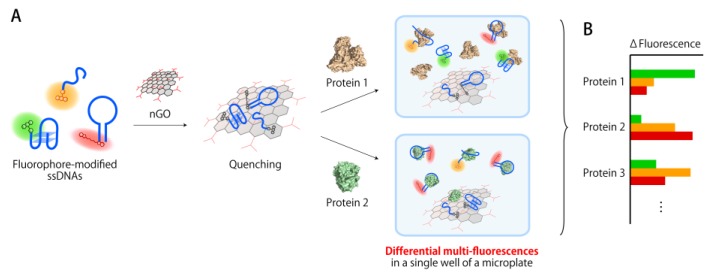
Schematic representation of the multi-fluorescent ssDNAs/nGO sensor system presented in this study. (**A**) Structurally different ssDNAs that contain different fluorophores are initially quenched simultaneously by complexation with nGO. The conjugates subsequently interact with different proteins in different ways, (**B**) which results in the generation of multicolor fluorescence signatures.

We envisioned that fluorophore-modified single-stranded DNA (ssDNA) could be suitable for the construction of multi-fluorescent sensing systems, as virtually any sequence of ssDNA can be synthesized commercially in high purity and labeled with fluorophores at low cost. This accessibility and tunability should create the structurally-diverse sensing elements necessary for high levels of discrimination. Recently, Pei et al. [38] and our group [39] have developed arrays of fluorophore-modified ssDNA quenched via noncovalent complexation with nano-graphene oxide (nGO) for the identification of proteins based on measurements of spatially separated conjugates. Encouraged by these studies, we have used conjugates between nGO and three ssDNAs with different sequences and different fluorophores in order to construct a sensing system that affords unique protein fluorescence signatures from a single microplate well (Figure 1). In this system, three fluorophore-modified ssDNAs are quenched simultaneously via complexation with nGO (Figure 1A). The subsequent addition of proteins causes a disruption of the conjugates via competitive interactions with individual ssDNAs, and in some cases with nGO. The use of sequentially- and structurally-diverse ssDNAs results in the unique release of ssDNAs from the conjugates due to the different binding affinities between the individual ssDNAs and the proteins. A multichannel fluorescence reading then allows generating signatures that reflect the amount of released ssDNA (Figure 1B). Data interpretation through pattern recognition methods demonstrated that this system shows high levels of discrimination for a variety of proteins.

## 2. Materials and Methods 

### 2.1. Materials

Nano-graphene oxide (nGO; width = 90 ± 15 nm; thickness = 1 nm) dispersed in water was obtained from EM Japan Co., Ltd (Tokyo, Japan). ssDNAs labeled with carboxyfluorescein (FAM) at the 3′ terminus (**P1**-FAM), with carboxytetramethylrhodamine (TAMRA) at the 3′ terminus (**P2**-TAMRA), or with indodicarbocyanine (Cy5) at the 5′ terminus (**P3**-Cy5) were synthesized and purified by Eurofins Genomics (Ebersberg, Germany). Pepsin from porcine stomach (Pep), β-galactosidase from *Escherichia coli* (Gal), albumin from bovine serum (BSA), catalase from bovine liver (Cat), transferrin from human serum (Tra), myoglobin from equine heart (Myo), α-chymotrypsinogen from bovine pancreas (Chy), lysozyme from hen egg white (Lys), and cytochrome c from horse heart (Cyt) were obtained from Sigma Chemical Co. (St. Louis, MO, USA). Immunoglobulin G from human serum (IgG) was obtained from Equitech-Bio, Inc. (Kerrville, TX, USA). Phosphate-buffered saline (PBS) was obtained from Wako Pure Chemical Ind. (Osaka, Japan).

### 2.2. Fluorescence Quenching Study

Fluorescence measurements were performed on a Spectra max GEMINI XPS (Molecular devices, Sunnyvale, CA, USA). Solutions (200 μL) containing 20 nM **P1**-FAM, 20 nM **P2**-TAMRA, 20 nM **P3**-Cy5, and 0–160 µg/mL nGO in PBS buffer (pH = 7.4) were prepared in each well of a 96-well plate (96-well black flat-bottom polystyrene NBS microplates; Corning Inc., Corning, NY, USA) using a PIPETMAX system (Gilson Inc., Middleton, WI, USA). After incubation (*T* = 30 °C, *t* = 10 min), fluorescence spectra were recorded at *T* = 30 °C using three different excitation wavelengths (**P1**-FAM: λ_ex_ = 480 nm, λ_em_ = 520–595 nm; **P2**-TAMRA: λ_ex_ = 530 nm, λ_em_ = 575–665 nm; **P3**-Cy5: λ_ex_ = 630 nm, λ_em_ = 660–750 nm). Binding isotherms were produced based on changes in the fluorescence intensity at λ_em_ = 519 nm (**P1**-FAM), 579 nm (**P2**-TAMRA), and 664 nm (**P3**-Cy5).

### 2.3. Signature-Based Sensing

Solutions (180 μL) containing 22.2 nM **P1**-FAM, 22.2 nM **P2**-TAMRA, 22.2 nM **P3**-Cy5, and 111.1 µg/mL nGO in PBS buffer (pH = 7.4) were prepared in each well of a 96-well plate using a PIPETMAX system. After incubation (*T* = 30 °C, *t* = 10 min), the fluorescence intensities were collected at seven different channels (vide infra). Subsequently, aliquots (20 μL) of 150 µg/mL proteins in PBS (pH = 7.4) were added to each well, before the fluorescence intensities were recorded after incubation (*T* = 30 °C, *t* = 10 min). For the sensing of protein in the presence of interferents, human serum that was diluted 3000-fold with PBS (pH = 7.4) was used as a solvent. This process was repeated six times to generate a training data matrix consisting of 7 channels × 6 replicates. The raw data matrix was processed using linear discriminant analysis (LDA) and hierarchical clustering analysis (HCA) in SYSTAT 13 (Systat Software Inc., San Jose, CA, USA). For a blind test, the same process was repeated six times to generate a test data matrix. The test data were classified into groups generated by the training matrix according to their shortest Mahalanobis distances.

## 3. Results and Discussion

### 3.1. Construction of a Multi-Fluorescent ssDNAs/nGO Sensor 

**Figure 2:**
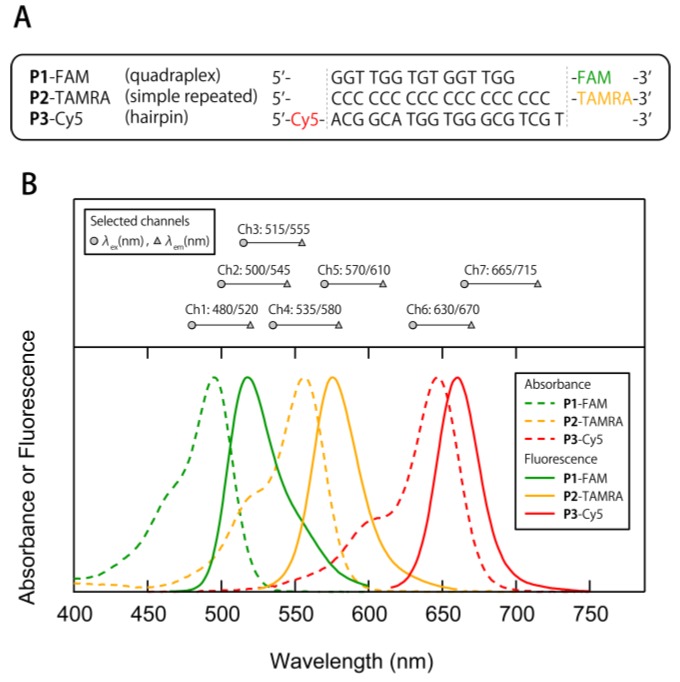
Absorption and fluorescence properties of the fluorophore-modified ssDNAs **P1**-FAM, **P2**-TAMRA, and **P3**-Cy5. (**A**) Sequence of the three ssDNAs and the modified fluorophores. (**B**) Normalized UV-VIS absorption and emission spectra of **P1**-FAM, **P2**-TAMRA, and **P3**-Cy5 in PBS (pH = 7.4). The λ_ex_/λ_e__m_ combinations to acquire fluorescence signatures are shown in the upper panel.

To construct a multi-fluorescent ssDNAs/nGO sensor, we designed three fluorophore-modified ssDNAs (Figure 2A); **P1**-FAM: a quadraplex-formative sequence with FAM (λ_ex max_/λ_em max_ = 495 nm/518 nm); **P2**-TAMRA: a simple repeated sequence with TAMRA (λ_ex max_/λ_em max_ = 555 nm/575 nm); **P3**-Cy5: a hairpin-structure-formative sequence with Cy5 (λ_ex max_/λ_em max_ = 645 nm/660 nm). These ssDNAs bear different sequences, and two of these can fold into different higher-order structures, which were expected to impart the individual elements of the sensor system with differential cross-reactivity [38,39]. In addition, well-separated absorption and emission spectra allow the readout of independent emissions of the fluorophores (Figure 2B).

**Figure 3:**
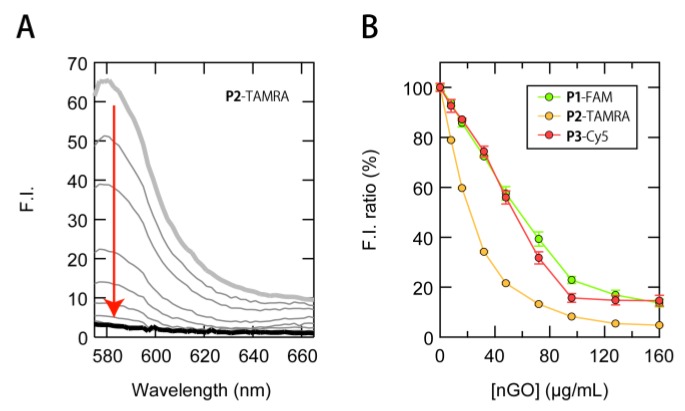
Preparation of ssDNAs/nGO conjugates. (**A**) Changes in the emission spectra corresponding predominantly to **P2**-TAMRA (*λ*_ex_ = 535 nm) and (**B**) the fluorescence ratio of 20 nM fluorophore-modified ssDNAs in the presence of different concentrations of nGO in PBS (pH = 7.4); **P1**-FAM: λ_ex_ = 480 nm, *λ*_em_ = 519 nm; **P2**-TAMRA: λ_ex_ = 535 nm, λ_em_ = 579 nm; **P3**-Cy5: λ_ex_ = 630 nm, λ_em_ = 664 nm.

Initially, a fluorescence titration of nGO was carried out on an equimolar mixture of the three ssDNAs (20 nM) to examine whether nGO is able to quench the fluorescence of the ssDNAs simultaneously. For instance, the fluorescence emission of **P2**-TAMRA can be observed dominantly when excited at 535 nm and detected at 579 nm. As shown in Figure 3A, the addition of nGO to a solution containing the three ssDNAs resulted in a concentration-dependent quenching of **P2**-TAMRA. Although **P1**-FAM and **P3**-Cy5 showed a similar pronounced decrease in fluorescence emission (Figure 3B and Figure S1), the corresponding quenching efficacies were lower than that of **P2**-TAMRA. This may be attributed to the shielding of nucleobases in **P1**-FAM and **P3**-Cy5, caused by the DNA folding, which could hamper *π*-*π* stacking interactions with nGO [40]. Therefore, in the following sensing experiments we used a binding ratio that provides high fluorescence quenching for all ssDNAs and minimal reproducible responses with the addition of 15 µg/mL proteins (Figure S2), i.e., 20 nM ssDNAs and 100 µg/mL nGO.

### 3.2. Multi-Fluorescent Signature-Based Protein Sensing

Subsequently, we tested the ability of the multi-fluorescent ssDNAs/nGO sensor to generate fluorescence signatures of proteins. For that purpose, ten proteins that vary in size and surface charges were chosen as sensing targets (Table 1). Each protein solution (20 µL) in PBS (pH = 7.4) was mixed with solutions (180 µL) of ssDNAs/nGO conjugates in PBS (pH = 7.4) to reach a final concentration of 15 µg/mL protein in a 96-well microplate. The fluorescence signals from individual wells were recorded as (*I*–*I*_0_) at seven different channels (Figure 2B), generating a data matrix of 7 channels × 10 proteins × 6 replicates (Table S1). Four channels provided almost independent emissions of **P1**-FAM (Ch1), **P2**-TAMRA (Ch4), and **P3**-Cy5 (Ch6 and Ch7). Conversely, the other three channels (Ch2, Ch3, and Ch5) were likely located between the absorption and emission spectra of two of the three fluorophores, which should allow investigating the effectiveness of using spectral crosstalk.

**Figure 4:**
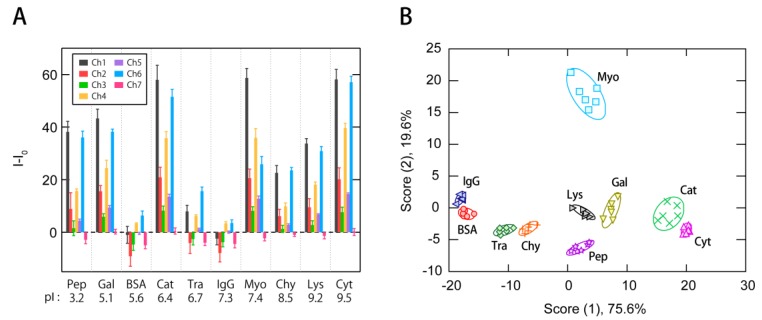
Protein identification using the multichannel ssDNAs/nGO sensing system presented in this study. (**A**) Signatures of changes in the fluorescence intensity upon addition of protein solutions (15 µg/mL) from a sensor consisting of 100 µg/mL nGO and 20 nM of the three fluorophore-modified ssDNAs. Values are shown as mean values ± SD (*n* = 6). (**B**) Discriminant score plot for protein solutions (15 µg/mL) obtained from the seven-channel system, whereby ellipsoids represent confidence intervals (±1 SD) for the individual analytes.

The thus-obtained fluorescence signatures (Figure 4A) likely show good reproducibility for the analyte proteins. These signatures were then subjected to an LDA in order to examine whether the individual signatures differ significantly. LDA is a supervised pattern recognition algorithm that provides a graphical output that offers insight into the clustering of the data and information on the classification ability [4]. A linear discriminant score plot revealed ten well-separated clusters corresponding to the individual proteins (Figure 4B). In this plot, each point represents the fluorescence signature of a single analyte protein. The first discriminant score, i.e., Score (1), provided the best discrimination among the classes, which accounted for 75.6% of the total variance. We expected basic proteins such as Lys (pI = 9.2) and Cyt (pI = 9.5) to exhibit a higher binding affinity than neutral or acidic proteins, as both nGO and the ssDNAs are negatively charged at pH = 7.4. However, the first discriminant scores showed almost no correlation with the pIs of the proteins (*r* = −0.17). Considering the equally low correlation with the protein size (*r* = 0.18), the sum of interactions regarding various characteristics, such as electrostatic and aromatic properties, hydrophobicity, surface heterogeneity and morphology, may possibly be responsible for the output as fluorescence signatures.

Then, a leave-one-out cross-validation analysis, the so-called jackknife classification procedure [41], was performed to determine the classification potential of the multi-fluorescent ssDNAs/nGO conjugate sensor. Using a single channel afforded classification accuracies of 50%, 35%, 33%, 63%, 75%, 70%, and 33% for Ch1 to Ch7, respectively, while the accuracy increased to 97% when using all seven channels (Table 2). Thus, it can be concluded that the sensor can acquire sufficient information to discriminate a variety of proteins from a single well. This system was able to detect ten different proteins at 15 µg/mL, ranging from 32 nM (Gal) to 1.3 µM (Cyt), which is comparable to the performance of a previously reported multi-fluorescent signature-based protein sensor [33]. It should be noted that using merely three channels that are selective to individual fluorophores (Ch1, Ch4 and Ch6) afforded a comparable classification accuracy (98%; Table 2), while a partial overlap between confidence ellipses was observed in the discriminant score plot (Figure S3). The accuracy for IgG did not reach 100% in all cases shown in Table 2, possibly due to the lower responses of ssDNAs/nGO conjugates compared to other proteins (Figure 4A). The slight increase in accuracy for BSA upon decreasing the number of channels may be attributed to the higher levels of noise in Ch2, Ch3, Ch5, and Ch7.

Thereafter, we used 60 newly-prepared samples for a blind test, and the new cases were assigned to proteins according to their shortest Mahalanobis distances. Only four samples were misclassified when using seven channels, affording a classification accuracy of 93% (Table S2). The accuracy only slightly decreased to 88% when using merely Ch1, Ch4, and Ch6 (Table S2). These results suggest that it should be important to read out individual ssDNAs independently in the discrimination of proteins. It is possible that the high contributions of Ch1, Ch4, and Ch6 for protein discrimination is partly due to the higher magnitude in response compared to other channels (Figure 4A).

### 3.3. Exploraion of Effective Sensing Channels for the Discrimination of Proteins

**Figure 5:**
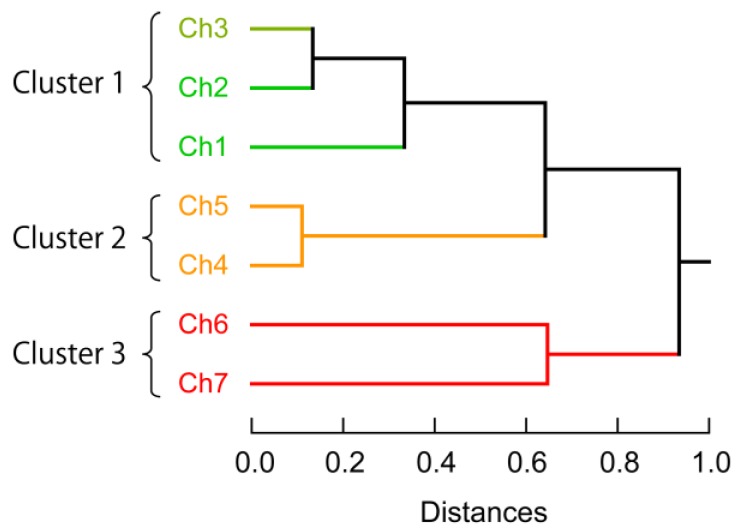
Clustering analysis of the discriminative channels of the multi-fluorescent ssDNAs/nGO sensor presented in this study. A hierarchical clustering dendrogram was created based on the Euclidean distances using the Ward method and a dataset of 7 channels × 10 analytes × 6 replicates. **P1**-FAM is primarily excited by Ch1 and Ch2; **P2**-TAMRA is primarily excited by Ch4 and Ch5; **P3**-Cy5 is primarily excited by Ch6 and Ch7.

In order to gain further insight into the effective selection of channels, we investigated the relevance of individual channels on the generation of fluorescence signatures using HCA, which determines clusters on the basis of the Euclidean distances between elements of a dataset. Therein, each channel was standardized prior to the analysis based on the following equation: *z* = (*x* − *μ*)/*σ*, wherein *z* is the standardized score, *x* the raw response (*I*–*I*_0_), *μ* the mean value of the population, and *σ* the standard deviation of the population. Three clusters were observed (Figure 5), i.e., cluster 1 includes Ch1–Ch3, cluster 2 includes Ch4 and Ch5, while cluster 3 includes Ch6 and Ch7. This result indicates a low correlation between channels included in each cluster. As estimated from Figure 2B, Ch2 and Ch5 primarily read out the fluorescence of **P1**-FAM and **P2**-TAMRA, respectively. Hence, each cluster corresponds most likely to individual fluorophore-modified ssDNAs, suggesting that the use of different sequences and higher-order structures of ssDNA induce diverse cross-reactivity, which is a key feature for the generation of differential signatures. In their entirety, these results suggest that acquiring independent emissions of **P1**-FAM, **P2**-TAMRA, and **P3**-Cy5 is critical to design accurate multi-fluorescent sensing systems, which is consistent with the results from the Jackknife classification and the blind test (Table 2 and Table S2).

Note that the properties of nGO should be considered to construct sensing systems with higher discrimination capability, as the interactions between nGO and proteins may play a partial role in the generation of fluorescence signatures. Given the recent progress in GO research, it has not only become possible to produce GO at lower costs and on a larger scale [42,43], but also to control its size, defects, and surface functionality [44,45,46]. As GO with different characteristics interact differently with human cells and proteins [44,46], an optimization of these characteristics should improve the discrimination capability of the system.

### 3.4. Protein Sensing in the Presence of Human Serum

**Figure 6:**
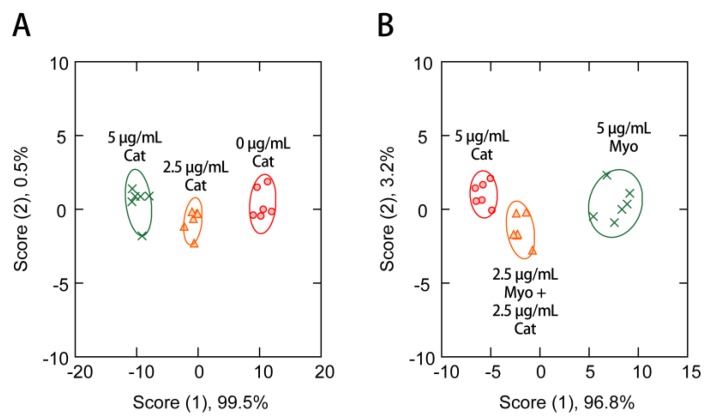
Discriminant score plot for (**A**) different concentrations of Cat (0–5 μg/mL), as well as (**B**) samples containing Cat and/or Myo with a total concentration of 5 μg/mL in the presence of human serum obtained from the seven-channel system consisting of 100 µg/mL nGO and 20 nM of the three fluorophore-modified ssDNAs, whereby ellipsoids represent confidence intervals (±1 SD) for the individual analytes.

The performance of this sensing system was further evaluated for the identification of two different proteins (Cat and Myo) in the presence of interferent human serum. An estimated >10,000 proteins are present in human serum [47], generating a challenging, complex matrix. It has been suggested that serum levels of Cat [48] and Myo [49,50] could potentially be used as biochemical markers for particular diseases. Using the seven-channel system, 100% discrimination accuracy based on the jackknife classification was achieved for different concentrations of Cat (0–5 μg/mL) (Figure 6A), and samples containing Cat and/or Myo with a total concentration of 5 μg/mL (Figure 6B). Cat clusters moved along the x-axis with increasing concentration (Figure 6A), while the 1:1 mixture of Cat and Myo was located between these components (Figure 6B). These results indicate the potential of this method for the detection of proteins in solutions containing complex interferents.

## 4. Conclusions

We have developed a multi-fluorescent ssDNAs/nGO sensor for the discrimination of proteins. Using conjugates between nGO and three ssDNAs that differ with respect to the sequence and fluorophore, various proteins were successfully identified based on their fluorescence signatures generated from a single microplate well. This system can be easily tuned and extended, as sensor elements with the following properties are commercially available: (i) ssDNAs with different structures and functions; (ii) fluorophores with different optical properties. The accessibility and tunability of this sensing system stands in stark contrast to previously reported multichannel signature-based sensing systems that require laborious synthetic [33] and/or protein expression protocols [34,35,36,37]. Due to the high discriminatory power and simple format, the sensor system presented herein should represent a highly promising tool for a facile and fast characterization of proteins or protein mixtures.

## Figures and Tables

**Table 1:** Properties of the proteins used in this study as sensing targets.

Protein	Source	Abbreviation	*M_w_*	pI
Pepsin	Porcine stomach	Pep	35,000	3.2
β-Galactosidase	*Escherichia coli*	Gal	465,000	5.1
Albumin	Bovine serum	BSA	66,000	5.6
Catalase	Bovine liver	Cat	230,000	6.4
Transferrin	Human serum	Tra	75,000	6.7
Immunoglobulin G	Human serum	IgG	143,000	7.3
Myoglobin	Equine heart	Myo	18,000	7.4
α-Chymotrypsinogen	Bovine pancreas	Chy	26,000	8.5
Lysozyme	Hen egg white	Lys	14,000	9.2
Cytchrome c	Horse heart	Cyt	12,000	9.5

**Table 2:** Classification accuracy of the multichannel ssDNAs/nGO sensor presented in this study.

Selected Channels							%Correct										
Ch1	Ch2	Ch3	Ch4	Ch5	Ch6	Ch7	BSA	Cat	Chy	Cyt	Gal	IgG	Lys	Myo	Tra	Pep	Total
							50	0	100	0	83	50	83	17	100	17	50
							67	17	50	17	83	33	17	0	67	0	35
							67	17	17	33	67	33	50	17	33	0	33
							50	0	100	67	67	67	83	0	100	100	63
							67	0	100	83	100	83	100	50	83	83	75
							67	67	67	83	83	83	67	33	100	50	70
							50	83	33	17	17	17	33	50	0	33	33
							100	100	100	100	100	83	100	100	100	100	98
							83	100	100	100	100	83	100	100	100	100	97

## Supplementary Materials

The following supplementary material is available online at www.mdpi.com/1424-8220/17/10/2194/s1, Table S1: Dataset matrix for the differences between the fluorescence intensity before and after the addition of 15 μg/mL of proteins generated from the multichannel ssDNAs/nGO system, Table S2: Blind test of 60 samples using the multichannel ssDNAs/nGO system, Table S3: Dataset matrix for the differences between the fluorescence intensity before and after the addition of different concentrations of Cat in the presence of human serum, generated from the multi-fluorescent nGO/ssDNA sensor, Table S4: Dataset matrix for the differences between the fluorescence intensity before and after the addition of Cat, Myo, and a 1:1 mixture (*w*/*w*) in the presence of human serum, generated from the multi-fluorescent nGO/ssDNA sensor, Figure S1: Changes in the emission spectra of P1-FAM and P3-Cy5 (20 nM fluorophore-modified ssDNA) in the presence of different concentrations of nGO in PBS (pH = 7.4), Figure S2: Fluorescence recovery of ssDNAs quenched with nGO upon addition of various concentrations of BSA, Figure S3: Sensing of proteins using the three-channel ssDNAs/nGO system.
