# Supplementary material for: A Multi-Fluorescent DNA/Graphene Oxide Conjugate Sensor for Signature-Based Protein Discrimination
Source: Sensors (Basel). 2017 Sep 23;17(10):2194. doi: 10.3390/s17102194 (PMC5677448; doi:10.3390/s17102194)
Supplement: Supplementary file 1 [file sensors-17-02194-s001.pdf]

Supporting Information for

A multi-fluorescent DNA/graphene oxide conjugate sensor for signature-based protein discrimination

Shunsuke Tomita,[1*] Sayaka Ishihara,[1] and Ryoji Kurita[1,2]*

1. Biomedical Research Institute, National Institute of Advanced Industrial Science and Technology, and DAILAB, 1-1-1 Higashi, Tsukuba, Ibaraki 305-8566, Japan.
2. Faculty of Pure and Applied Sciences, University of Tsukuba, 1-1-1 Tennodai, Tsukuba, Ibaraki 305-8573, Japan.

*Correspondence: s.tomita@aist.go.jp (S.T.); r.kurita@aist.go.jp (R.K.); Tel.: +81-29-861-2634 (S.T.)

Table S1. Dataset matrix for the differences between the fluorescence intensity before and after the addition of 15 μg/mL proteins generated from the multi-fluorescent nGO/ssDNA sensor.

Analyte	Ch1	Ch2	Ch3	Ch4	Ch5	Ch6	Ch7
Cat	64.40	25.38	10.34	36.91	14.01	55.06	1.25
Cat	58.59	23.41	9.06	33.41	12.84	52.74	0.97
Cat	64.18	22.84	8.36	39.84	14.49	54.11	0.71
Cat	54.53	15.17	5.56	34.40	12.20	49.28	-1.91
Cat	55.68	20.99	9.37	36.49	14.40	49.24	0.70
Cat	50.49	17.52	6.80	33.51	13.14	48.50	1.17
Gal	41.56	16.84	6.37	21.18	8.62	38.48	0.44
Gal	41.33	17.65	6.88	21.05	8.46	37.86	1.74
Gal	41.06	13.84	5.42	23.69	9.36	37.00	0.49
Gal	40.31	12.13	3.90	24.57	9.09	37.36	-0.92
Gal	48.72	17.08	6.58	27.95	10.39	39.23	-0.42
Gal	46.77	15.99	6.49	27.72	10.14	39.41	0.22
Myo	61.96	25.37	9.73	34.53	12.36	22.35	-1.15
Myo	55.58	21.77	8.68	30.92	11.75	22.45	-1.84
Myo	60.99	20.71	7.82	37.68	13.13	29.13	-2.01
Myo	53.29	14.85	5.22	33.61	11.71	25.39	-3.97
Myo	62.08	21.54	9.18	40.75	14.37	28.59	-2.54
Myo	57.97	19.02	8.09	38.03	13.17	27.30	-2.23
pep	43.69	16.43	4.48	15.90	4.83	38.71	-1.99
pep	42.54	16.58	5.55	15.45	4.66	38.87	-0.38
pep	34.39	2.31	-1.33	14.58	3.76	32.84	-4.20
pep	34.94	4.30	-0.57	15.18	3.58	34.10	-4.47
pep	37.82	7.06	0.92	16.72	5.09	35.69	-3.13
pep	35.56	6.71	0.19	16.48	4.49	36.02	-2.90
Lys	36.90	13.69	4.54	18.22	7.07	33.60	0.04
Lys	34.53	12.60	4.18	16.75	6.66	31.82	-0.35
Lys	32.61	8.11	1.84	17.46	6.56	30.41	-1.96
Lys	31.31	4.42	0.06	17.59	6.29	28.50	-3.13
Lys	33.07	9.29	3.36	19.16	6.86	29.70	-1.31
Lys	33.98	9.06	2.52	19.44	7.06	30.96	-1.37

Chy	22.04	8.17	1.74	8.65	2.31	23.13	-0.49
Chy	20.12	7.71	2.79	8.22	2.62	23.32	-0.61
Chy	22.47	3.84	0.25	10.18	2.70	22.91	-1.64
Chy	19.19	1.80	-0.96	9.36	2.45	22.20	-1.44
Chy	25.97	7.68	2.16	11.62	3.18	24.66	-1.38
Chy	25.76	7.35	1.72	11.06	3.43	24.97	-0.04
Cyt	64.38	27.31	10.70	39.34	14.65	60.04	1.63
Cyt	60.75	23.59	9.03	37.88	13.99	57.97	1.31
Cyt	56.38	16.13	5.63	39.53	14.33	54.98	-2.01
Cyt	53.67	16.96	6.13	37.53	14.07	53.85	-0.54
Cyt	57.32	18.79	7.26	42.12	15.21	57.93	0.28
Cyt	56.18	17.98	6.89	41.28	14.85	57.58	0.04
Tra	11.01	0.24	-0.75	6.38	1.41	17.54	-2.79
Tra	8.80	0.33	0.03	5.76	1.31	17.15	-2.32
Tra	5.45	-7.61	-4.33	5.80	0.66	14.14	-4.97
Tra	4.96	-9.12	-5.85	5.87	0.86	13.64	-4.59
Tra	8.81	-5.43	-2.88	6.86	1.67	15.93	-4.50
Tra	8.70	-2.95	-1.72	6.65	1.18	15.47	-4.44
BSA	3.33	-4.60	-2.28	3.49	0.16	8.82	-3.80
BSA	0.03	-4.61	-1.72	3.28	0.08	6.92	-4.54
BSA	-1.39	-11.78	-6.39	3.55	-0.60	6.34	-3.58
BSA	-6.02	-14.09	-7.34	2.67	-0.98	3.83	-7.28
BSA	0.82	-9.86	-4.84	3.47	-0.53	7.16	-5.18
BSA	-2.53	-9.74	-5.31	3.08	-0.51	4.97	-5.35
IgG	1.14	-3.11	-1.64	3.95	0.45	5.90	-2.64
IgG	-4.93	-6.16	-2.34	2.26	0.45	3.01	-2.77
IgG	-2.88	-9.19	-4.82	3.61	-0.07	3.35	-5.12
IgG	-4.93	-13.41	-6.51	3.28	-0.74	2.29	-6.59
IgG	-1.29	-7.72	-3.75	3.86	0.22	3.49	-4.85
IgG	-1.39	-7.75	-3.42	3.60	0.18	2.97	-4.52

Table S2. Blind test of 60 samples using the multi-fluorescent nGO/ssDNA sensor.

Identification	Ch1	Ch2	Ch3	Ch4	Ch5	Ch6	Ch7	Seven channels		Three channels (Ch1, Ch4, and Ch6)	
								Verification	Accuracy	Verification	Accuracy
Cat	62.75	22.78	9.75	45.79	16.93	47.41	-1.51	Cat	YES	Cat	YES
Cat	49.32	16.81	7.34	36.98	14.43	45.44	0.46	Cat	YES	Cat	YES
Cat	64.37	24.04	9.89	45.40	17.52	53.76	0.68	Cat	YES	Cat	YES
Cat	59.00	20.34	7.28	43.28	16.07	54.11	1.22	**Cyt**	**NO**	**Cyt**	**NO**
Cat	67.81	26.70	10.56	44.11	16.67	56.21	2.23	Cat	YES	Cat	YES
Cat	58.28	19.68	8.10	38.68	15.01	54.61	0.51	Cat	YES	**Cyt**	**NO**
Gal	41.19	14.84	6.47	30.53	12.26	33.51	-0.22	Gal	YES	Gal	YES
Gal	32.87	9.98	4.40	24.93	10.49	31.61	-1.31	Gal	YES	Gal	YES
Gal	36.38	13.22	4.99	25.21	9.77	33.39	-0.46	Gal	YES	Gal	YES
Gal	33.65	11.04	4.60	24.37	9.87	32.17	0.55	Gal	YES	Gal	YES
Gal	41.42	16.26	6.80	30.89	12.20	37.44	0.61	Gal	YES	Gal	YES
Gal	37.07	13.83	6.33	28.42	11.92	36.82	0.59	Gal	YES	Gal	YES
Myo	64.04	24.80	10.90	43.51	16.07	27.48	-1.62	Myo	YES	Myo	YES
Myo	53.57	16.81	7.68	41.43	14.69	-2.37	-5.89	Myo	YES	Myo	YES
Myo	58.28	21.23	8.97	41.63	15.31	25.55	-1.45	Myo	YES	Myo	YES
Myo	58.96	21.35	10.33	44.02	15.90	-0.71	-4.49	Myo	YES	Myo	YES
Myo	64.88	25.55	11.23	45.03	16.87	24.10	-1.09	Myo	YES	Myo	YES
Myo	58.40	21.59	8.61	40.24	15.04	21.44	-2.53	Myo	YES	Myo	YES
pep	34.61	3.18	-1.24	16.29	4.29	29.67	-4.11	pep	YES	**Lys**	**NO**
pep	33.96	4.68	-1.12	15.80	4.06	30.61	-4.96	pep	YES	**Lys**	**NO**
pep	37.34	7.57	0.94	18.41	6.31	36.05	-0.53	**Lys**	**NO**	pep	YES
pep	36.40	8.50	1.25	18.04	5.75	36.53	-1.11	pep	YES	pep	YES
pep	39.28	9.04	1.54	15.94	5.06	35.75	-3.33	pep	YES	pep	YES
pep	38.69	12.30	3.18	15.83	5.35	37.13	-2.21	pep	YES	pep	YES
Lys	28.10	6.27	1.97	19.69	7.47	25.93	-2.42	Lys	YES	Lys	YES
Lys	27.68	2.59	0.67	19.94	6.53	24.07	-3.78	Lys	YES	Lys	YES
Lys	31.71	10.26	3.61	19.28	7.62	30.75	-0.52	Lys	YES	Lys	YES
Lys	29.84	7.70	2.38	18.99	7.49	29.26	-1.45	Lys	YES	Lys	YES
Lys	33.18	10.80	3.18	20.26	8.24	31.05	-0.25	Lys	YES	Lys	YES
Lys	33.04	10.24	3.13	19.76	7.68	31.50	-0.66	Lys	YES	Lys	YES
Chy	17.69	1.40	0.16	10.20	3.21	17.56	-2.03	Chy	YES	**Tra**	**NO**
Chy	18.46	2.54	0.21	11.76	3.60	18.63	-1.62	Chy	YES	Chy	YES
Chy	18.56	4.11	1.14	10.84	3.70	20.89	-1.09	Chy	YES	Chy	YES
Chy	18.51	5.84	1.69	10.95	3.13	20.58	-0.43	Chy	YES	Chy	YES
Chy	21.30	5.20	1.99	12.24	4.05	23.48	-1.12	Chy	YES	Chy	YES
Chy	18.91	6.04	1.68	11.46	4.11	23.59	-0.21	Chy	YES	Chy	YES
Cyt	47.00	11.44	3.95	31.27	11.03	41.74	-2.93	**Gal**	**NO**	**Gal**	**NO**
Cyt	57.22	20.98	8.65	44.08	16.82	56.85	2.01	Cyt	YES	Cyt	YES
Cyt	55.91	20.85	8.52	43.86	16.55	56.08	1.68	Cyt	YES	Cyt	YES
Cyt	58.18	22.46	9.54	40.63	15.37	56.84	0.88	Cyt	YES	Cyt	YES
Cyt	51.57	15.57	5.60	34.31	12.19	42.99	-1.40	**Gal**	**NO**	**Cat**	**NO**
Cyt	56.35	18.95	7.74	39.62	15.01	56.17	0.44	Cyt	YES	Cyt	YES
Tra	5.25	-6.35	-3.90	6.50	1.33	12.39	-5.43	Tra	YES	Tra	YES
Tra	5.82	-6.46	-3.75	7.07	1.31	12.26	-4.57	Tra	YES	Tra	YES
Tra	8.90	-3.84	-2.96	7.33	2.19	16.58	-3.05	Tra	YES	Tra	YES
Tra	7.89	-2.19	-1.78	6.88	1.94	16.11	-2.60	Tra	YES	Tra	YES
Tra	11.46	-0.30	-2.03	7.23	2.13	17.68	-2.30	Tra	YES	Tra	YES
Tra	10.25	-1.55	-1.51	6.87	1.92	16.51	-2.82	Tra	YES	Tra	YES
BSA	-0.57	-11.48	-6.07	3.93	0.24	5.65	-4.97	BSA	YES	BSA	YES

BSA	-3.82	-11.94	-6.63	3.06	-0.39	4.67	-5.77	BSA	YES	BSA	YES
BSA	1.80	-9.03	-4.53	3.76	0.34	7.61	-5.10	BSA	YES	BSA	YES
BSA	-1.62	-9.48	-4.76	3.13	-0.12	5.86	-3.50	BSA	YES	BSA	YES
BSA	1.40	-7.41	-4.62	3.29	0.24	7.42	-4.69	BSA	YES	BSA	YES
BSA	-1.11	-9.08	-5.06	2.78	-0.14	6.67	-4.70	BSA	YES	BSA	YES
IgG	-3.47	-10.77	-5.05	3.54	-0.28	1.79	-6.41	IgG	YES	IgG	YES
IgG	-4.75	-12.71	-5.55	3.44	0.05	1.36	-6.59	IgG	YES	IgG	YES
IgG	0.52	-6.76	-3.61	4.30	0.50	4.51	-3.65	IgG	YES	IgG	YES
IgG	-0.65	-9.02	-4.01	3.96	0.79	3.84	-3.75	IgG	YES	IgG	YES
IgG	2.91	-4.47	-2.75	4.47	0.78	5.90	-3.03	IgG	YES	IgG	YES
IgG	0.49	-5.81	-3.12	3.74	0.35	5.20	-4.57	IgG	YES	IgG	YES

Table S3. Dataset matrix for the differences between the fluorescence intensity before and after the addition of different concentrations of Cat in the presence of human serum generated from the multi-fluorescent nGO/ssDNA sensor.

Analyte	Ch1	Ch2	Ch3	Ch4	Ch5	Ch6	Ch7
0 μg/mL Cat	7.98	-0.08	0.22	2.29	-0.42	4.60	-7.24
0 μg/mL Cat	8.38	0.92	0.69	2.60	-0.71	6.44	-5.75
0 μg/mL Cat	8.15	1.39	0.80	2.52	0.03	6.88	-4.63
0 μg/mL Cat	6.54	-1.77	-0.06	2.44	-1.00	1.53	-7.08
0 μg/mL Cat	6.39	-0.66	0.29	2.22	-0.74	3.49	-6.20
0 μg/mL Cat	6.21	-0.36	0.63	2.00	-0.95	4.45	-5.96
2.5 μg/mL Cat	11.99	2.32	1.54	5.25	1.22	9.54	-5.97
2.5 μg/mL Cat	9.04	1.64	1.11	4.59	0.56	9.42	-5.80
2.5 μg/mL Cat	10.09	2.36	1.26	4.52	1.03	10.68	-3.77
2.5 μg/mL Cat	9.92	1.49	1.20	5.45	1.05	7.19	-5.33
2.5 μg/mL Cat	9.48	1.38	0.82	5.09	0.39	8.42	-5.93
2.5 μg/mL Cat	9.70	1.97	1.43	4.73	0.55	7.93	-4.61
5 μg/mL Cat	14.06	2.80	1.97	7.43	2.39	11.92	-4.41
5 μg/mL Cat	13.28	3.67	1.82	7.42	1.86	13.27	-4.30
5 μg/mL Cat	12.60	3.68	2.21	6.54	1.74	11.76	-2.71
5 μg/mL Cat	13.06	2.22	2.04	7.48	1.85	10.11	-4.43
5 μg/mL Cat	11.65	3.22	1.57	6.99	1.65	11.25	-5.03
5 μg/mL Cat	12.86	2.28	1.81	7.06	1.74	11.32	-4.87

Table S4. Dataset matrix for the differences between the fluorescence intensity before and after the addition of Cat, Myo, and a 1:1 (w/w) mixture of Cat and Myo in the presence of human serum, generated from the multi-fluorescent nGO/ssDNA sensor.

Analyte	Ch1	Ch2	Ch3	Ch4	Ch5	Ch6	Ch7
5 μg/mL Myo	13.13	2.80	1.99	6.98	2.16	-18.45	-9.10
5 μg/mL Myo	13.51	4.01	1.97	6.89	1.89	-6.11	-6.81
5 μg/mL Myo	12.38	3.22	2.03	6.11	1.70	-5.02	-6.30
5 μg/mL Myo	13.50	2.38	2.20	8.46	2.09	-36.83	-12.00
5 μg/mL Myo	12.07	2.83	1.80	7.06	1.51	-29.98	-10.91
5 μg/mL Myo	12.18	2.93	2.16	6.65	1.57	-27.30	-9.29
5 μg/mL Cat	15.80	5.37	2.61	8.46	2.68	12.82	-2.11
5 μg/mL Cat	15.87	5.60	2.60	9.32	2.65	15.39	-4.51
5 μg/mL Cat	17.01	5.54	2.94	8.92	3.06	14.83	-2.45
5 μg/mL Cat	15.11	2.39	2.73	9.68	3.03	12.59	-4.18
5 μg/mL Cat	14.64	4.89	2.59	9.29	2.49	14.24	-4.74
5 μg/mL Cat	13.95	3.16	2.44	8.89	2.61	12.53	-4.68
Mixture of Cat and Myo	15.06	3.01	2.55	8.42	2.79	13.57	-3.43
Mixture of Cat and Myo	14.10	4.85	2.28	7.74	2.27	14.20	-4.21
Mixture of Cat and Myo	13.40	3.89	2.16	7.32	2.43	14.66	-2.96
Mixture of Cat and Myo	13.86	2.59	1.81	7.91	2.16	11.31	-4.62
Mixture of Cat and Myo	12.33	3.59	1.95	7.76	2.00	12.82	-5.16
Mixture of Cat and Myo	13.58	2.91	2.23	7.72	2.12	12.48	-2.77

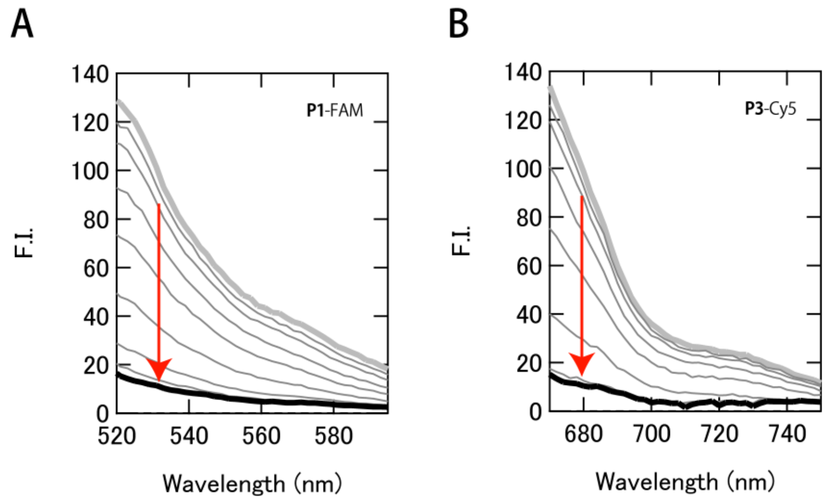

Figure S1. Changes in the emission spectra of (A) **P1**-FAM (λ_{ex} = 535 nm) and (B) **P3**-Cy5 (λ_{ex} = 630 nm) (20 nM fluorophore-modified ssDNA) in the presence of different concentrations of nGO in PBS (pH = 7.4).

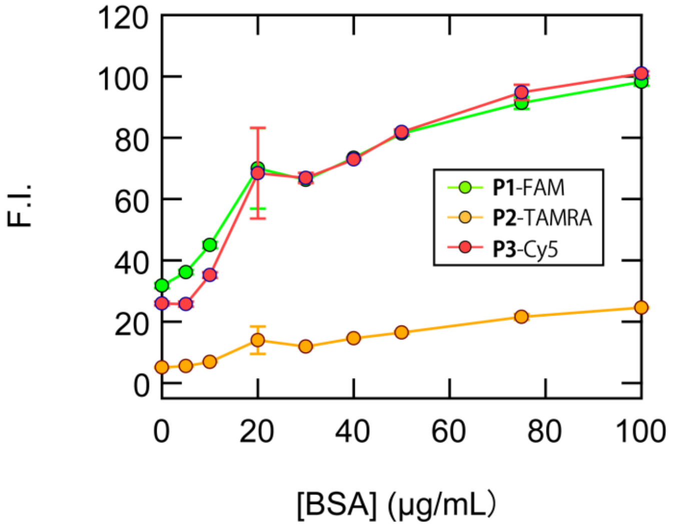

Figure S2. Fluorescence recovery of ssDNAs (20 nM) quenched with 100 μg/mL nGO upon addition of various concentrations of BSA in PBS (pH = 7.4); **P1**-FAM: λ_{ex} = 480 nm, λ_{em} = 519 nm; **P2**-TAMRA: λ_{ex} = 535 nm, λ_{em} = 579 nm; **P3**-Cy5: λ_{ex} = 630 nm, λ_{em} = 664 nm.

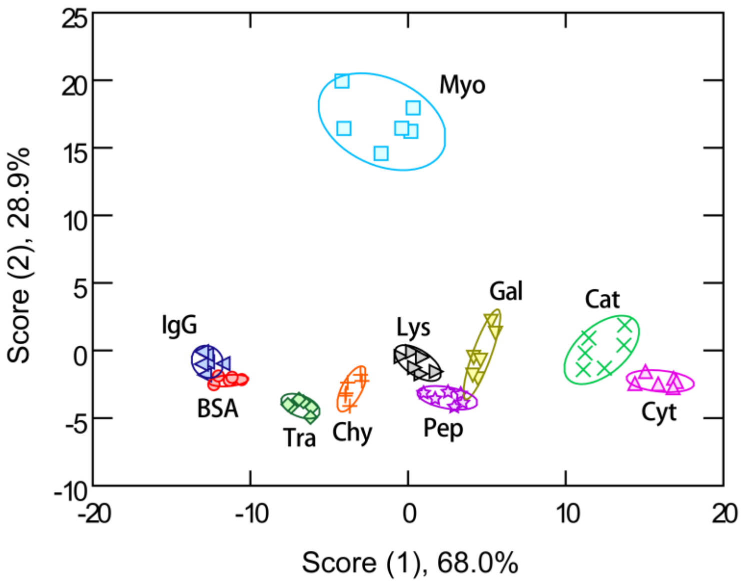

Figure S3. Discriminant score plot for 15 μg/mL proteins obtained from the three-channel nGO/ssDNA system (Ch1, Ch4, and Ch6), whereby ellipsoids represent confidence intervals (± 1 SD) for the individual analytes.
